# Epidemiology of Reemerging Scarlet Fever, Hong Kong, 2005–2015

**DOI:** 10.3201/eid2310.161456

**Published:** 2017-10

**Authors:** Chun Fan Lee, Benjamin J. Cowling, Eric H. Y. Lau

**Affiliations:** World Health Organization Collaborating Centre for Infectious Disease Epidemiology and Control, University of Hong Kong, Hong Kong, China

**Keywords:** scarlet fever, group A streptococcus, GAS, Streptococcus pyogenes, bacteria, bacterial infection, epidemiology, seasonality, kindergarten, Hong Kong

## Abstract

Annual incidence of scarlet fever in Hong Kong remained elevated after an upsurge in 2011. Incidence increased from 3.3/10,000 children <5 years of age during 2005–2010 to 18.1/10,000 during 2012–2015. Incidence was higher among boys and was 32%–42% lower in the week following school holidays.

Scarlet fever, caused by group A *Streptococcus* (*Streptococcus pyogenes*), was a common infectious disease in children before the early 20th century (*1*) and a major cause of death, with a case-fatality risk >30% (*2*,*3*). Various factors, including improvements in hygiene and the introduction of effective antibiotics, led to the disappearance of scarlet fever as a major cause of pediatric deaths by the mid-20th century (*4*). However, reemerging cases of scarlet fever were reported in China in 2011 and the United Kingdom in 2014 (*5*,*6*). Hong Kong also experienced an upsurge in scarlet fever cases in 2011, with a >10-fold increase over the previous incidence rate (*7*,*8*). The reason for the surge is unclear. One report has suggested that toxin acquisition and multidrug resistance might have contributed (*9*). Since 2011, the reported number of scarlet fever cases in these locations has remained at elevated levels (*10*,*11*). We analyzed the patterns in scarlet fever incidence in Hong Kong during 2005–2015, including the upsurge since 2011.

## The Study

**Figure 1:**
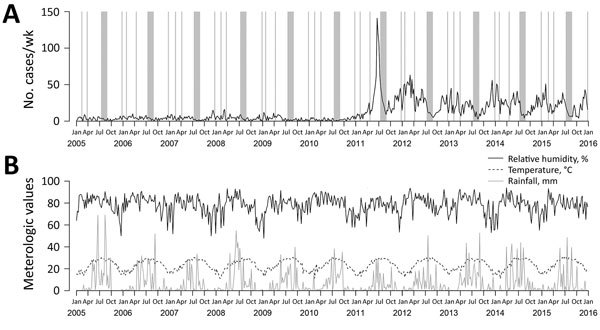
Trends of scarlet fever cases and meteorologic variables affecting reemergence of scarlet fever, Hong Kong, 2005–2015. A) Weekly number of notified scarlet fever cases. Gray bars indicate periods of school holidays. B) Weekly average of temperature, relative humidity, and rainfall.

Scarlet fever is a notifiable disease in Hong Kong. We collected individual data, including age, sex, dates of illness onset, and travel history, from 7,266 local case-patients <14 years of age (with 3,304 having laboratory-confirmed cases) reported to the Department of Health during 2005–2015. The 2011 upsurge was characterized by a sharp peak (Figure 1, panel A). During that year, 1,438 cases (incidence 17.5/10,000 children <14 years of age) were reported, exceeding the total number of 1,117 cases (average incidence 2.1/10,000 children <14 years of age) in the previous 6 years (2005–2010). Since then, the annual number of reported cases has remained at a relatively high level, with an average of 14.5 cases/10,000 children <14 years of age during 2012–2015. The elevated pattern was more apparent in children <5 years of age; among this age group, annual incidence averaged 3.3/10,000 children during 2005–2010, jumped to 23.9/10,000 in 2011, and dropped slightly to 18.1/10,000 during 2012–2015.

We investigated the effect of various epidemiologic and meteorologic variables on scarlet fever incidence by using hierarchical multivariable negative binomial regression, accounting for autocorrelation and annual and biannual seasonal trends by using Fourier terms (i.e., including sine and cosine terms in the model) (*12*). Specifically, we modeled the age- and sex-specific weekly number of scarlet fever cases by age and sex of the patient; linear time trend; average temperature, relative humidity, and rainfall in the previous week (Figure 1, panel B); and school holidays in the previous week. We divided age into 4 groups reflecting the type of school attended: 0–2 years (nursery), 3–5 years (kindergarten), 6–11 years (primary school), and 12–14 years (junior high school). In Hong Kong, school holidays include Christmas (≈2 weeks), Chinese New Year (≈10 days), Easter (≈10 days), and summer vacation (≈7 weeks) (Figure 1, panel A). We included the logarithm of the population size for each age–sex group as an offset term. We first fitted a model with age, sex, and a linear time trend variable for the whole study period, allowing for change in slope after the upsurge, and found a significant change in the linear time trend after the 2011 upsurge (p<0.001). Therefore, we divided the time-series into preupsurge (2005–2010 [311 weeks]) and postupsurge (2012–2015 [209 weeks]) periods, excluding data in 2011 as a window period of transition.

**Figure 2:**
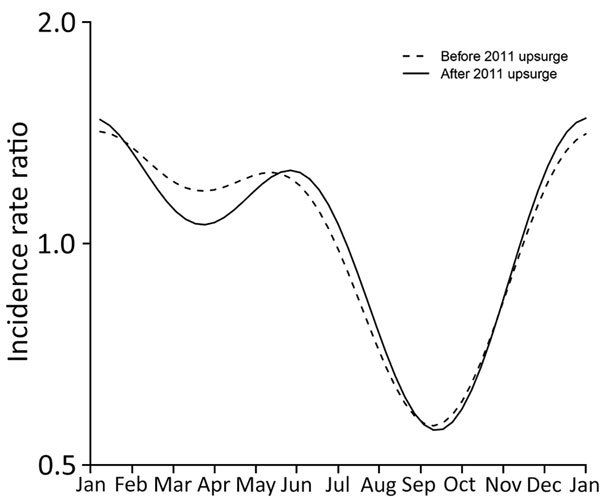
Estimated incidence rate ratios of the seasonal component from the negative binomial regression models before and after the 2011 upsurge of scarlet fever, Hong Kong. Both curves show a bimodal pattern with peak incidence during December–January and May–June and lowest incidence in September.

We then added seasonality to the model in each period (model I; Technical Appendix Table). Seasonal trends were similar in both periods, with a more pronounced bimodal pattern after the upsurge (Figure 2). The trough ended in early September, when school begins, then disease activity increased to its peak in January, followed by a milder peak in June. Previous studies have indicated a similar bimodal seasonal pattern (*6*,*13*).

The final model also included the variables of school holidays and meteorologic factors in the preceding week (Table). This model fitted the scarlet fever incidence satisfactorily, especially for the age groups of 3–5 years and 6–11 years (Technical Appendix Figure). We observed no significant linear trend in scarlet fever incidence before the upsurge but a mild decreasing trend after the upsurge (adjusted incidence rate ratio [IRR] 0.92; 95% CI 0.89–0.94) of an 8% decrease per year. In general, boys had a higher IRR than girls at all age groups, and children 3–5 years of age had the highest IRR, followed by those 6–11 years of age and then those 0–2 years of age, for both sexes. We found a significant age–sex interaction in the postupsurge period. Specifically, boys had a higher risk for scarlet fever than girls, with an adjusted IRR of 1.08 (95% CI 0.87–1.34) at 0–2 years of age, 1.47 (95% CI 1.32–1.65) at 3–5 years of age, 1.31 (95% CI 1.18–1.46) at 6–11 years of age, and 2.01 (95% CI 1.26–3.20) at 12–14 years of age. Similar to what has been reported elsewhere (*5*,*13*), almost all reported cases in Hong Kong were among children. The relatively high incidence among children of kindergarten age corresponds to the start of schooling, consistent with studies in China (*14*). The new cohort of children in kindergarten might partly drive the major winter peak. Boys being more at risk than girls might be attributable to more physical interactions or poorer personal hygiene among boys (*15*).

**Table:** Estimated incidence rate ratios of various epidemiologic and meteorological factors affecting reemergence of scarlet fever, Hong Kong, 2005–2015*

Variable	IRR (95% CI)

Preupsurge period, 2005–2010, 311 wks		Postupsurge period, 2012–2015, 209 wks
Linear time trend, per year	0.98 (0.95–1.02)	0.92 (0.89–0.94)
Sex			
F	1.00		1.00
M	1.33 (0.92–1.94)		1.08 (0.87–1.34)
Age group, y			
0–2	1.00		1.00
3–5	2.78 (2.02–3.90)		3.17 (2.63–3.84)
6–11	1.29 (0.95–1.80)		2.06 (1.71–2.50)
12–14	0.13 (0.07–0.24)		0.14 (0.09–0.21)
Sex × age interaction			
Boys, 0–2 y	1.00		1.00
Boys, 3–5 y	1.11 (0.72–1.68)		1.36 (1.07–1.74)
Boys, 6–11 y	1.07 (0.70–1.61)		1.22 (0.96–1.55)
Boys, 12–14 y	1.01 (0.44–2.36)		1.86 (1.12–3.15)
School holidays in the preceding week	0.68 (0.55–0.85)		0.58 (0.51–0.65)
Temperature, °C	0.991 (0.953–1.031)		0.963 (0.940–0.987)
Relative humidity, %	0.981 (0.972–0.990)		0.997 (0.992–1.002)
Rainfall, mm	1.009 (1.002–1.016)		0.998 (0.993–1.002)

*Incidence rate ratios estimated by negative binomial regression models also accounting for autocorrelation and annual and biannual seasonality using Fourier terms with periods of 1 year (T) and half a year (T/2), where T = 365.25/7 weeks. IRR, incidence rate ratio.

In 2011, the outbreak reached its peak with 141 cases in the second half of June but sharply fell to 9 cases in the last week of August, at the end of the summer vacation (Figure 1, panel A). However, a clear upturn could be observed once the new school year started. We observed a similar pattern in each subsequent year. Also, school holidays were significantly associated with lower incidence, with IRRs of 0.68 (95% CI 0.55–0.85) before the upsurge (a 32% reduction) and 0.58 (95% CI 0.51–0.65) after the upsurge (a 42% reduction). Together with the observation that the prenursery-age children had a lower incidence, school is probably a major transmission site of scarlet fever because children began to increase their social contact substantially.

In the preupsurge period, relative humidity (adjusted IRR 0.981, 95% CI 0.972–0.990) and rainfall (adjusted IRR = 1.009; 95% CI 1.002–1.016) were significantly associated with incidence of scarlet fever, whereas temperature was not significantly associated. In contrast, in the postupsurge period, temperature had a significant effect on scarlet fever incidence (adjusted IRR 0.963; 95% CI 0.940–0.987), whereas relative humidity and rainfall had an insignificant effect.

## Conclusions

Scarlet fever cases continued to occur in Hong Kong at elevated incidence rates for 5 consecutive years after a major epidemic in 2011. Scarlet fever incidence is higher among younger children entering schools and during school days. School-based control measures, especially for boys 3–5 years of age, could be particularly important in scarlet fever control. A limitation of our study is that we relied on reported cases to study scarlet fever epidemiology, and subclinical infections might have occurred. Moreover, some uncaptured or unobserved factors not considered in this study might have influenced the trend observed. Further community-based studies, including serologic studies, might further elucidate the epidemiology of this reemerging disease.

Technical Appendix Models estimated with hierarchical multivariable negative binomial regression to characterize reemergence of scarlet fever and observed and predicted numbers of scarlet fever cases, Hong Kong, 2005–2015.
