# Supplementary material for: Epidemiology of Reemerging Scarlet Fever, Hong Kong, 2005–2015
Source: Emerg Infect Dis. 2017 Oct;23(10):1707–10. doi: 10.3201/eid2310.161456 (PMC5621532; doi:10.3201/eid2310.161456)
Supplement: Technical Appendix — Models estimated with hierarchical multivariable negative binomial regression to characterize reemergence of scarlet fever and observed and predicted numbers of scarlet fever cases, Hong Kong, 2005–2015. [file 16-1456-Techapp-s1.pdf]

Article DOI: https://doi.org/10.3201/eid2310.161456

Epidemiology of Reemerging Scarlet Fever, Hong Kong, 2005–2015

Technical Appendix.

Technical Appendix Table. Various models estimated using hierarchical multivariable negative binomial regression to characterize reemergence of scarlet fever, Hong Kong, 2005–2015*

	IRR (95% CI)			
	Model I: investigation of seasonality		Model II: full model	
Variable	Before 2011 upsurge (2005–2010)	After 2011 upsurge (2012–2015)	Before 2011 upsurge (2005–2010)	After 2011 upsurge (2012–2015)
Linear time trend, per year	0.98 (0.95–1.02)	0.91 (0.88–0.94)	0.98 (0.95–1.02)	0.92 (0.89–0.94)
Sex				
F	1.00	1.00	1.00	1.00
M	1.33 (0.92–1.94)	1.07 (0.87–1.34)	1.33 (0.92–1.94)	1.08 (0.87–1.34)
Age group, y				
0–2	1.00	1.00	1.00	1.00
3–5	2.76 (2.00–3.88)	3.02 (2.50–3.67)	2.78 (2.02–3.90)	3.17 (2.63–3.84)
6–11	1.28 (0.94–1.79)	1.94 (1.60–2.37)	1.29 (0.95–1.80)	2.06 (1.71–2.50)
12–14	0.13 (0.07–0.24)	0.15 (0.09–0.22)	0.13 (0.07–0.24)	0.14 (0.09–0.21)
Sex × age interaction				
Boys, 0–2 y	1.00	1.00	1.00	1.00
Boys, 3–5 y	1.10 (0.72–1.68)	1.33 (1.04–1.70)	1.11 (0.72–1.68)	1.36 (1.07–1.74)
Boys, 6–11 y	1.06 (0.69–1.60)	1.20 (0.94–1.53)	1.07 (0.70–1.61)	1.22 (0.96–1.55)
Boys, 12–14 y	1.01 (0.44–2.36)	1.86 (1.12–3.16)	1.01 (0.44–2.36)	1.86 (1.12–3.15)
School holidays in the preceding week			0.68 (0.55–0.85)	0.58 (0.51–0.65)
Temperature, °C			0.991 (0.953–1.031)	0.963 (0.940–0.987)
Relative humidity, %			0.981 (0.972–0.990)	0.997 (0.992–1.002)
Rainfall, mm			1.009 (1.002–1.016)	0.998 (0.993–1.002)

*Incidence rate ratios estimated in both models were also accounted for autocorrelation and annual and biannual seasonality using Fourier terms with periods of 1 year (T) and half a year (T/2), where T = 365.25/7 weeks. IRR, incidence rate ratio.

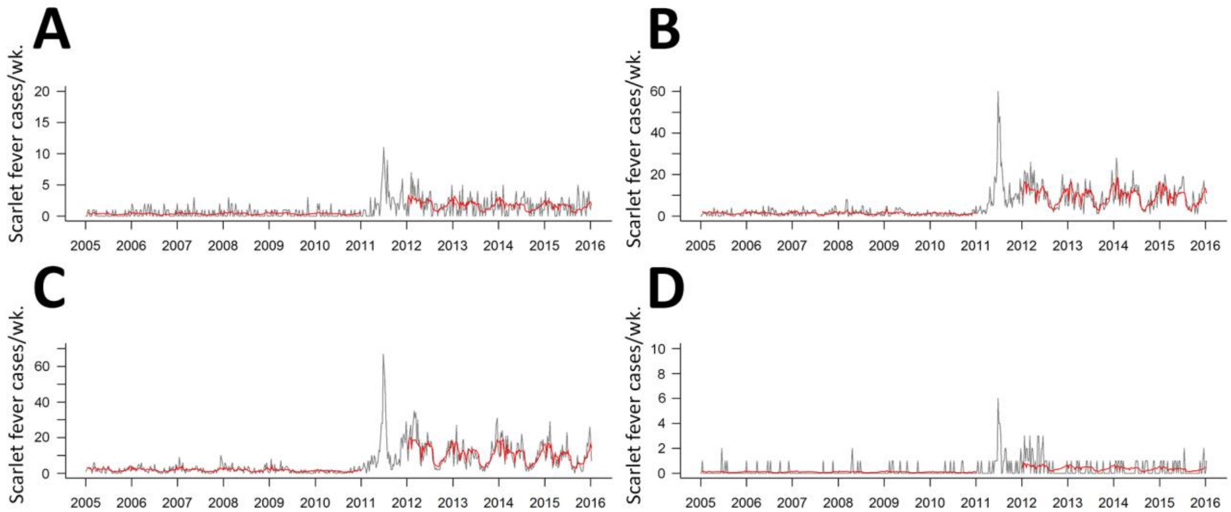

Technical Appendix Figure. Observed (gray) and predicted (red) numbers of scarlet fever cases in Hong Kong during 2005–2015. A) Children aged 0–2 years. B) Children aged 3–5 years. C) Children aged 6–11 years. D) Children aged 12–14 years. The transition to higher scarlet fever incidence occurred in 2011, and data from this period were excluded from the analysis.
